# Bidirectional causal relationship between obesity and osteoarthritis: Insights from a two-sample Mendelian randomization study

**DOI:** 10.1016/j.ocarto.2025.100636

**Published:** 2025-05-27

**Authors:** Anne Kamps, Jos Runhaar, Katerina Trajanoska, William D. Thompson, Weiya Zhang, Bahar Sedaghati-khayat, Joyce B.J. van Meurs, Sita M.A. Bierma-Zeinstra

**Affiliations:** a Department of General Practice, Erasmus MC University Medical Center Rotterdam, the Netherlands; b Department of Internal Medicine, Erasmus MC University Medical Center Rotterdam, the Netherlands; c Canada Excellence Research Chair in Genomic Medicine, Victor Philip Dahdaleh Institute of Genomic Medicine, Department of Human Genetics, Faculty of Medicine and Health Sciences, McGill University, Montreal, QC, Canada; d School of Medicine, Faculty of Medicine & Health Sciences, University of Nottingham, Nottingham, United Kingdom; e Department of Orthopedics and Sports Medicine, Erasmus MC University Medical Center Rotterdam, the Netherlands

## Abstract

**Objective:**

Osteoarthritis (OA) is a prevalent chronic disease associated with disability worldwide, and obesity is a key modifiable risk factor for OA. The study's aim was to investigate the causal relationship between obesity and OA.

**Method:**

This study employed a two-sample Mendelian randomization (MR) approach to investigate the bidirectional causal relationship between obesity, using body mass index (BMI) as its proxy, and OA of the knee, hip, and hand. Genetic instruments were derived from large-scale GWAS meta-analyses, including ∼681,000 individuals for BMI and ∼827,000 individuals (177,000 OA cases) for OA. Inverse variance weighted with multiplicative random effects analysis was performed as primary analysis, and in addition sensitivity analyses relying on different assumptions were performed.

**Results:**

The MR analysis revealed that genetically predicted BMI had a causal effect on increased risk of knee (OR 1.91, 95 ​% CI 1.80–2.03), hip (OR 1.52, 95 ​% CI 1.41–1.64) and hand OA (OR 1.21, 95 ​% CI 1.04–1.23). Sensitivity analyses confirmed the robustness of these associations. However, there was no evidence for a causal effect from knee, hip or hand OA on BMI.

**Conclusion:**

This study provides strong evidence supporting a causal effect of obesity (measured by BMI) on OA, with a more pronounced effect in weight-bearing knee & hip joints compared to non-weight-bearing hand joint. There was no causal evidence for the reverse direction. Future research could look more in depth into differences in the genetic variants that may represent different biological underlying mechanisms.

## 1 Introduction

Osteoarthritis (OA) is a chronic and progressive joint disease. It is the most common form of arthritis and is most often diagnosed in the knee, hip, and hand joints. Due to chronic pain and function limitation, OA is an important and growing contributor to the total number of years lived in disability worldwide [1].

Extensive evidence links OA to obesity, which is considered one of the most important modifiable risk factors for the condition [2]. A meta-analysis indicates that obesity accounts for up to 50 ​% of the population-attributable fraction, representing the percentage reduction in disease occurrence if the specific risk factor were eliminated [3]. Besides a risk factor, obesity is also considered a target for therapy. Weight loss interventions exhibit modest to moderate therapeutic and prognostic effects on the progression and severity of OA symptoms [4]. For instance, a randomized controlled trial conducted in obese patients with knee OA demonstrated positive effects of weight loss on joint load, inflammation, and clinical outcomes like pain and function [5]. Biological mechanisms that mediate the relationship between obesity and OA include chronic low-grade systemic inflammation (such as circulating inflammatory cytokines), and metabolic dysregulation (such as higher levels of risk altering lipids), which may contribute to cartilage degradation and synovial inflammation independently of mechanical loading [6,7].

The development of OA occurs over an extended period, making it less feasible to investigate the causal influence of modifiable risk factors, such as obesity, in an appropriate prospective study design. Although conventional observational studies are more practicable, they may be susceptible to biases such as residual confounding or reverse causation. These biases may lead to under- or overestimation of the impact of obesity due to unmeasured (synergistic) effects of for example comorbidities, lifestyle factors, and limited mobility, which can further augment the risk of OA. Mendelian randomization (MR) is a causal inference method based on instrumental variable (IV) analysis that reduces confounding and reverse causation, offering a more viable alternative to RCTs [8]. MR uses genetic variants as proxies for modifiable exposures to identify causal effects on disease outcomes, with obesity as the exposure and OA as the outcome in this study.

Several MR studies have investigated the causal association between obesity and OA, the majority focusing on overall OA [9]. Most site-specific studies investigated knee or hip OA. For example, the risk of knee OA increased with odds ratios (ORs) between 1.69 and 2.00, and the risk of hip OA with ORs between 1.41 and 2.05 [10, 11]. Evidence for other joint sites is limited. For spine OA, sedentary behavior (more specifically watching television), was found to increase risk in a BMI-mediated pathway [12]. For ankle and foot OA, no direct causal associations with body mass index (BMI) have been reported, although increased BMI has been linked to a higher risk of ankle-foot injuries [13].

Only two studies have explored hand OA, yielding conflicting results [14,15]. Including hand OA is relevant as it is one of the most affected joints, and non-weight-bearing. Consequently, exploring the causal effects of obesity on hand OA can broaden our understanding of mechanisms underlying OA development beyond mechanical and weight-bearing influences. Furthermore, none of the previous studies examined causality in the reverse direction, i.e., if OA causally affects obesity. As a result, the presence of reverse causation in this context remained unresolved up to now.

In summary, this study addresses current gaps in the literature by investigating the bidirectional causal relationship between obesity and site-specific OA of the knee, hip, and hand. The hypothesis posits that there is a causal effect of obesity on OA, with the estimated effect size varying across joints due to disparities in weight-bearing versus non-weight-bearing joint loading. Furthermore, it is hypothesized that there is no causal effect of OA on obesity in all three examined joints.

## 2 Methods

**Fig. 1:**
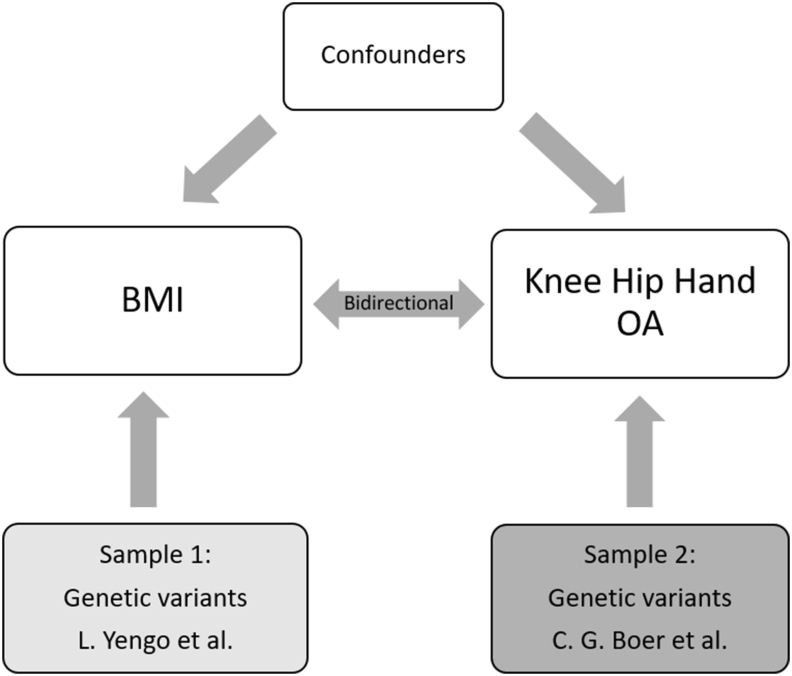
A directed acyclic graph illustrating the bidirectional, two-sample MR design of the study. In the first analyses, one sample is used to instrument BMI as the exposure and a second sample to instrument knee, hip, and hand OA (studied separately) as the outcomes. For the other direction, the exposure and outcomes are reversed, so that OA becomes the exposure and BMI the outcome.

A bidirectional, two-sample MR study was conducted. In a two-sample MR the association between the genetic variants and exposure and outcome are estimated in two independent datasets. This approach increases the sample size and enhances statistical power [16]. Bidirectional MR involves testing both the causal effects of the exposure on the outcome and vice versa (See Fig. 1).

### 2.1 Data source I: genetic variants for obesity

In the analysis where obesity was the exposure, BMI was utilized as a proxy for obesity. Genetic association estimates for BMI were obtained from a large meta-analysis that combined data from the UK Biobank (UKB) and the Genetic Investigation of Anthropometric Traits (GIANT) consortium [17]. BMI was defined as kilograms per square meter and was corrected for, among others, age, sex, and genotype-based principal components. BMI measurements in the GIANT consortium could be either assessed during visits or self-reported, while in the UKB, height and weight measurements were taken during assessment visits [18,19]. The total sample size of the meta-analysis was 681,275 individuals of European ancestry. Detailed information on the individual studies and their methods can be found in the respective original publications.

### 2.2 Data source II: genetic variants for OA

Genetic variants for OA were derived from a large-scale genome-wide association meta-analysis conducted across 13 international cohorts, encompassing up to 826,690 individuals (including 177,517 OA patients) predominantly of European ancestry [20]. In most cases, OA was self-reported by patients, although some cohorts defined OA through clinical diagnoses, such as coded diagnoses from electronic health records or used X-rays. The current study focused on three subtypes of OA: knee OA (including total knee replacement), hip OA (including total hip replacement), and hand OA (including finger and thumb OA), all as defined in the original study. Demographics, genotyping methods, and detailed definitions of the different OA phenotypes can be found in the supplementary materials of the original publication.

### 2.3 Instrumental variable selection

The IVs for the MR analyses were selected as single nucleotide polymorphisms (SNPs) associated with BMI at a genome-wide significance threshold (P ​< ​5 ​× ​10^−8^). To ensure independence, clumping was performed, retaining one representative SNP per linkage disequilibrium region with a threshold of r^2^ ​< ​0.001 within a 10000-kilobases (kb) window using the 1000G European reference panel. In cases where SNPs were missing in the outcome sample, proxies in high linkage disequilibrium (r^2^ ​> ​0.8) with the missing SNP were selected. Next, SNP-exposure and SNP-outcome coefficients were harmonized to ensure that effect estimates corresponded to the same allele. Palindromic SNPs with indeterminate allele frequencies (Minor allele frequency >0.49) were excluded from the analysis due to the inability to verify allele orientation [21]. Steiger filtering was applied to remove SNPs if their association with the outcome was stronger than their association with the exposure [22]. If a substantial number of SNPs (>10 ​%) were removed after harmonization and filtering, proxies were searched for again.

### 2.4 Statistical analyses

The primary causal estimates were obtained through a multiplicative inverse-variance weighted (IVW) random effect model, which, unlike fixed effect model, allows all SNPs to exhibit balanced horizontal pleiotropy [23]. In two-sample MR studies utilizing summarized data, it is recommended to report multiplicative IVW analysis as the primary analysis, as it accounts for heterogeneity in variant-specific causal estimates [24]. We did not apply a formal correction for multiple testing, given the related nature of the OA sites and the hypothesis-driven context of the analysis. As recommended by Burgess et al., an overly conservative approach to multiple testing is often unnecessary in MR studies, particularly when prior biological or epidemiological evidence supports the tested relationships [24]. Sensitivity analyses were conducted to compare the causal estimates obtained from the IVW method with those from methods relying on different assumptions that affect the method robustness to pleiotropy or genetic heterogeneity. These analyses are discussed in depth under the next subheading.

For each exposure-outcome association studied, the reported estimates included IVW, MR-Egger, weighted median, and MR-Pleiotropy RESidual Sum and Outlier (MR-PRESSO). Causal estimates of BMI on OA are reported as ORs with 95 ​% confidence intervals or *P*-values. The ORs represented the increased risk of having the outcome per one standard deviation increase in genetically predicted BMI. Causal estimates of OA on BMI represented the average change in BMI per 2.72-fold increase in the prevalence of OA (for example, an increase in prevalence from 1 to 2.72 ​%) [25]. MR analyses were performed using TwoSampleMR (v 0.5.6) in R (v 4.2.1). The study was conducted in accordance with the STROBE-MR guideline [26]. STROBE-MR checklist is provided in supplementary file S11.

### 2.5 Sensitivity analysis for violation of assumptions

To obtain unbiased MR estimates, several key assumptions of MR need to be fulfilled regarding the IVs: (1) Relevance: the IV and exposure are strongly associated; (2) Independence: the IV is not confounded with the outcome; (3) Exclusion restriction: the IV only affects the outcome through its effect on the exposure; (4) Homogeneity: the effect of the exposure is constant within levels of the IV & monotonicity: the IV affects the exposure only in one direction. The first three core assumptions are crucial for estimating valid causal effects [27].

Testing the first assumption involved calculating the instrument strength of the exposure represented by the F-statistic, estimated by the chi-square approximation [28]. A value of F ​> ​10 generally indicates a good instrument, although context and genetic instrument composition should be considered [29]. The second assumption relies on Mendel's second law of independent assortment, which states that genetic variants are randomly allocated during meiosis and therefore confounding factors are less likely to occur. While this assumption is usually fulfilled for environmental confounders, it may not be the case for biological confounders. Therefore, potential confounding factors were described in the discussion's *limitations* section. The third assumption can be violated in various ways, with horizontal pleiotropy being the most important concern. Horizontal pleiotropy occurs when genetic instruments not only influence the exposure of interest but also affect the outcome through other pathways. To detect this, the heterogeneity and asymmetry of the instruments for the exposure were assessed. Cochran's Q statistic was calculated to assess the presence and extent of heterogeneity. If Q was significantly larger than its degrees of freedom, it indicates heterogeneity among the IVs. The proportion of heterogeneity was then evaluated using I^2^ ([Cochran's Q – degrees of freedom]/Cochran's Q x 100 ​%). Funnel plots of effect estimates against their standard error helped identify the presence of directional pleiotropy (demonstrated by asymmetric funnel plots), versus balanced pleiotropy (demonstrated by symmetrical funnel plots). In addition, MR-Egger regression was employed to estimate the direction of horizontal pleiotropy (intercept) and a pleiotropy-robust estimate of the causal effect (slope). Subsequently, a weighted median analysis was performed. This estimator assumes that up to 50 ​% of the IVs are invalid [30]. Furthermore, MR-PRESSO analysis was conducted to identify and correct outliers by estimating the IVW linear regression estimate after outlier removal [31].

Finally, the additional fourth assumption, “homogeneity”, is typically not met because the effect of a trait or disease as an exposure often varies among individuals. Thus, the causal estimates in this study are based on the weaker assumption of “monotonicity."

## 3 Results

### 3.1 Obesity to OA

#### 3.1.1 Obesity instrument

An instrument for obesity was obtained by selecting SNPs associated with BMI from a GWAS, see method for detailed information [16]. Following clumping, 490 SNPs were identified as IVs for BMI. No variants were missing in the extraction of the selected SNPs in the outcome samples. After harmonization and Steiger filtering, less than 5 ​% of the variants were excluded. Consequently, datasets consisting of 474, 472, and 474 SNPs were obtained for knee, hip, and hand OA, respectively. The instrument strength measurements revealed an explained variance of ∼4.8 ​% and an F statistic of 76.9, similar for all three types of OA. The knee OA instrument exhibited moderate heterogeneity (I^2^ ​= ​45.9 ​%), but no evidence of horizontal pleiotropy, as indicated by an Egger intercept of 0.00 and a *P*-value of 0.82. The heterogeneity I^2^ for hip OA was also moderate with 41.5 ​% and minimal for hand OA with 23.7 ​%, both with no horizontal pleiotropy.

Supplementary file S1 contains funnel plots used to assess the symmetry of exposure instruments for the analyses, both from BMI to OA as the reverse direction.

#### 3.1.2 Results knee OA

The IVW analysis showed a strong causal effect of obesity on knee OA: OR 1.91 (95 ​% CI: 1.80–2.03).

#### 3.1.3 Results hip OA

The IVW estimate also supported a causal effect on hip OA: OR 1.52 (95 ​% CI: 1.41–1.64).

#### 3.1.4 Results hand OA

For hand OA, the IVW analysis showed a weaker but statistically significant effect: OR 1.21 (95 ​% CI: 1.04–1.23).

Supplementary file S2 contains scatterplots of the causal estimates of all MR analyses (IVW, weighted median, MR Egger, weighted mode, and simple mode) in both directions. In all cases, there were either no outliers or the MR-PRESSO outlier-corrected estimate did not deviate from the raw IVW estimate.

### 3.2 OA to obesity

#### 3.2.1 Knee OA instrument

For the instrument of knee OA in the current study, the threshold of genome-wide significance (i.e., <5 ​× ​10−8) was used and following clumping, 28 variants were selected. After extracting these SNPs from the outcome dataset, 22 SNPs (including 10 proxies) were found, and after harmonization and Steiger filtering, 19 SNPs remained. The explained variance was 0.2 ​%, and the F statistic was 36.8. Although substantial heterogeneity of 86.7 ​% was observed, no evidence of horizontal pleiotropy was found (Egger intercept ​= ​0.01, *P*-value ​= ​0.12).

#### 3.2.2 Results

The IVW estimate indicated no causal effect of knee OA on obesity: β ​= ​0.02 (95 ​% CI: 0.03 to 0.07).

#### 3.2.3 Hip OA instrument

After applying a genome-wide significance filter, followed by clumping, harmonization, and Steiger filtering, 29 SNPs were selected as IVs for hip OA, including 11 proxies. The explained variance was 0.4 ​%, and the F statistic was 46.5. Although substantial heterogeneity of 88.9 ​% was observed, no evidence of horizontal pleiotropy was found (Egger intercept ​= ​0.00, *P*-value ​= ​0.51).

#### 3.2.4 Results

The IVW analysis found no causal effect of hip OA on obesity: β ​= ​−0.01 (95 ​% CI: 0.04 to 0.02).

#### 3.2.5 Hand OA instrument

9 SNPs were selected as IVs. After harmonization and Steiger filtering, 6 SNPs were retained. The explained variance was 0.08 ​%, and the F statistic was 39.7. Heterogeneity was observed at 57.8 ​%, and no evidence of horizontal pleiotropy was found (Egger intercept ​= ​0.00, *P*-value ​= ​0.88).

#### 3.2.6 Results

The IVW estimate did not indicate a causal effect of hand OA on obesity. Supplementary file S2 contains scatterplots of the causal estimates of all MR analyses (IVW, weighted median, MR Egger, weighted mode, and simple mode) in both directions. In all cases, there were either no outliers or the MR-PRESSO outlier-corrected estimate did not deviate from the raw IVW estimate.

### 3.3 Leave-one-out and single-SNP analyses

In both directions leave-one-out and single-SNP analyses were performedLeave-one out analyses from obesity to OA show that no single SNPs were driving the association (Supplementary Fig. S3–S5). For the other direction, OA to obesity, see supplementary file S6 for the plots. Furthermore, the single-SNP analyses show the effect of all SNPs independently. See Supplementary Fig. S7–S9 for the analyses from obesity to OA, and supplementary file S10 for the plots in the other direction.

## 4 Discussion

### 4.1 Summary of main findings

Two-sample bidirectional MR was conducted to investigate the causal relationship between obesity and subtypes of OA across three joint locations. The results demonstrated a genetically predicted causal effect of obesity on all three types of OA. BMI, used as proxy for obesity, exhibited the strongest association with OA in weight-bearing joints such as the knee (OR 1.91) and hip (OR 1.52), but also showed an effect in the non-weight-bearing hand joint (OR 1.21). These associations were robust, supported by strong IVs and confirmed through sensitivity analyses.

In contrast, none of the OA types showed a causal effect on obesity. This suggests that there is likely no causal effect of OA on obesity. However, it is important to consider that the lower instrument strengths of OA subtypes, along with the presence of substantial heterogeneity, may have weakened the analyses in this direction.

### 4.2 Comparison with other studies

Large scale observational studies have demonstrated the risk of obesity on development of OA. For knee OA, a meta-analysis with 446,219 individuals showed an OR of 2.02 for overweight and of 3.81 for obesity [3]. A large electronic health record cohort study (>5.5 million individuals) demonstrated increased HRs for the effect of overweight (BMI 25–30) on knee, hip and hand OA of 2.00, 1.46, 1.22 respectively; similar to the MR estimates found in the present study [32].

While observational findings demonstrate strong associations, these do not imply causality. In contrast, MR allows for stronger causal inference by leveraging genetic instruments, which are less susceptible to confounding and reverse causation. A systematic review included all MR studies that investigated the effects of lifestyle-related risk factors on OA, published up to 2022 [9]. Seven studies examining the risk of BMI on OA were identified, mostly published between 2019 and 2021. The pooled causal effect of BMI on overall OA yielded a causal estimate (IVW OR 1.49) that was comparable to the estimates found in the current study. However, the meta-analysis had the limitation that 6/7 studies used SNPs from the UKB as the outcome for OA, leading to substantial sample overlap and potentially reduced statistical power. The meta-analysis also showed 99 ​% heterogeneity, likely due to pooling OA data from different joint locations and using different diagnostic definitions for OA (structural and self-reported). The IVW method does not tolerate heterogeneity in the genetic variants, risking biased estimates despite the use of a random effects model [23].

A more recent study investigated the causal effect of BMI on overall OA as the primary outcome, with site-specific (knee, hip, and spinal OA) and sex-specific analyses as secondary outcomes [15]. In a sensitivity analysis, hand OA was also investigated. Similar to the current study, the ORs were 1.88 for knee, 1.50 for hip, and 1.23 for hand OA. Furthermore, the study showed that there was a consistent causal effect in males and females, and finally that when accounting for the waist-to-hip ratio in a multi variable MR analysis BMI showed a similar causal effect on OA as in the primary analysis.

### 4.3 Strengths of study

A unique strength of this study was the bidirectional analysis, which had not been conducted in any of the aforementioned studies. By performing bidirectional MR with the appropriate use of Steiger filtering, i.e. excluding genetic variants as instruments that were more strongly associated with the outcome than the exposure, the potential bias of reverse causation could be assessed. Secondly, increasing research emphasizes the importance of distinguishing between different phenotypes of OA due to differences in risk factors and comorbidity patterns, among other factors [33]. A major strength of this study was its investigation of separate subtypes of OA, including weight-bearing and non-weight-bearing joint subtypes. An advantage of using the comprehensive GO consortium meta-analysis was the ability to robustly demonstrate a causal effect of BMI on hand OA. The current study hereby confirmed the priory found causal effect of BMI on hand OA by L. Zhang et al. [15] and supports the hypothesis that obesity may cause OA through metabolic pathways too, rather than solely through biomechanical pathways.

Furthermore, all analyses were conducted with an appropriate primary analysis (multiplicative random-effects IVW) and were tested by several additional analyses that relied on weaker assumptions, such as the median weighted method. MR-Egger was used to assess pleiotropy, and no evidence of horizontal pleiotropy was found, confirming the assumption of good instrument strength. Finally, MR-PRESSO was employed to detect and correct the IVW estimate for potential outliers.

### 4.4 Limitations

Several limitations potentially impacted the analyses of this study. Firstly, the instruments for the OA subtypes had lower power compared to those for BMI, as indicated by the F statistic and explained variance. This might have limited the ability to detect causal effects of OA on BMI. Therefore, the absence of a statistically significant estimate should not be interpreted as an absolute absence of a causal effect.

Secondly, significant heterogeneity was observed in the analyses from OA to obesity. This heterogeneity may be attributed to differences in the genetic architecture of the two traits, variety in the definition of OA (for example hip OA defined by total hip replacement surgery versus self-reported) or confounding factors in the study design. Also, from obesity to OA there was up to 50 ​% heterogeneity. However, given that there is no evidence of unbalanced pleiotropy the results are less likely to be biased [34]. Because genetic variants that instrument BMI might have distinct biological mechanisms that could reasonably lead to different causal estimates, some level of heterogeneity might be expected in this direction. For example, a higher BMI can lead to differences in biomechanical joint loading, but when accompanied by a higher fat mass it can also lead to low-grade inflammation.

Using BMI as a proxy in MR studies has additional limitations. It assumes a linear relationship between BMI and the outcome, which may not accurately reflect the underlying biological mechanisms. BMI lacks specificity as it fails to differentiate between fat mass and lean mass or capture fat distribution within the body. One prior study showed that the causal effect of BMI on OA was not substantially affected when corrected for waist-to-hip ratio, but future studies could explore others measures that better capture body composition to improve BMI as a proxy for obesity [15].

Furthermore, the study's population predominantly consisted of individuals of European descent, limiting the generalizability of findings to other populations where the causal relationship between BMI and the outcome may vary due to genetic factors.

A final limitation is the participant overlap between the exposure and outcome datasets. Approximately one-third of the cases from the OA GWAS were drawn from the UKB, while the BMI GWAS included around 2/3 of the total amount of participants from the UKB, based on data from the GIANT consortium. Sample overlap between these GWASs can bias MR estimates, particularly in the presence of weak instruments. However, the genetic instruments used were strongly associated especially with BMI, which reduces the risk of weak instrument bias. In addition, a previous study using the same GWAS datasets conducted sensitivity analyses excluding overlapping samples and found no difference in causal estimates [15]. This suggests that the potential impact of sample overlap on our results is likely limited.

Despite these limitations, this study provides valuable insights into potential causal relationships between BMI and OA.

## 5 Conclusion

This study utilizes bidirectional MR to demonstrate a strong causal effect of obesity on knee, hip, and hand OA while finding no evidence of a causal effect of OA on BMI. The study's robust IVs and sensitivity analyses support the role of BMI in metabolic pathways contributing to OA risk, in addition to the better known biomechanical pathway. An important direction for future research is the use of multivariable Mendelian Randomization to investigate potential mediators such as metabolic and inflammatory markers. This approach could help clarify the biological mechanisms underlying the relationship between obesity and OA.

## Studies involving humans or animals

Clinical trials or other experimentation on humans must be in accordance with the ethical standards of the responsible committee on human experimentation (institutional and national) *and* with the Helsinki Declaration of 1975, as revised in 2000. Randomized controlled trials should follow the Consolidated Standards of Reporting Trials (CONSORT) guidelines and be registered in a public trials registry.

Studies involving experiments with animals were in accordance with institution guidelines Please sign below to certify your manuscript complies with the above requirements and then upload this form at https://www.editorialmanager.com/oac/

## Author contributions

SBZ and WZ wrote the funding application for the overarching, 10.13039/501100014034FOREUM funded ComOA project. In this application the conceptualization for this study (work package 4 of the project) took place. AK updated/finalized the statistical analysis plan, with the help and expertise of KT, JvM, BS and WT. AK performed the analyses, with supervision of JR and KT. The first results and their interpretation were discussed with KT, after which changes and/or additional analyses were performed by AK. All authors discussed the updated results and interpretation. AK drafted the manuscript and JR carried out the first round of revisions. All authors have read and contributed to the critical revision of the final version of the manuscript. AK takes responsibility for the integrity of the work as a whole, from inception to finished article.

## Data statement

The statistical code is available upon request. All data used is publicly available and original articles are referred to in the manuscript. Figures and tables of the raw results are all published in supplementary files.

## Authorship

All authors should have made substantial contributions to all of the following: (1) the conception and design of the study, or acquisition of data, or analysis and interpretation of data, (2) drafting the article or revising it critically for important intellectual content, (3) final approval of the version to be submitted. By signing below each author also verifies that he confirms that neither this manuscript, nor one with substantially similar content, has been submitted, accepted or published elsewhere (except as an abstract). Each manuscript must be accompanied by a declaration of contributions relating to sections (1), (2) and (3) above. This declaration should also name one or more authors who take responsibility for the integrity of the work as a whole, from inception to finished article. These declarations will be included in the published manuscript.

## Role of the funding source

This work was supported by the 10.13039/501100014034Foundation for Research in Rheumatology (10.13039/501100014034FOREUM) through a grant (2019–2022). FOREUM is a non-for-profit organization and did not participate in the design and conduct of the study; collection, management, analysis, and interpretation of the data; or preparation, review, or approval of the manuscript and the decision to submit the manuscript for publication. Link to grant webpage: https://www.foreum.org/comorbidities_oa.cfm?projectid=159.

## Declaration of competing interest

The authors declare the following financial interests/personal relationships which may be considered as potential competing interests:

A Kamps reports financial support was provided by foundation FOREUM. If there are other authors, they declare that they have no known competing financial interests or personal relationships that could have appeared to influence the work reported in this paper.

## Glossary

Mendelian randomization (MR): using measured variation in genes to examine the causal effect of an exposure on an outcome.
Two-sample MR.: the association between the genetic variants and exposure and outcome are estimated in two independent datasets.
Bidirectional MR.: both the causal effects of the exposure on the outcome and vice versa are tested.
Genetic variant.: an alteration in the most common DNA sequence; for example a deletion, insertion or translocation of one or more nucleotides.
Single nucleotide polymorphism: a commonly used type of genetic variant in MR where one nucleotide (A, C, G or T) has changed in a DNA sequence.

## Footnotes

Appendix A Supplementary data to this article can be found online at https://doi.org/10.1016/j.ocarto.2025.100636.
