# Supplementary figures and images for: Bidirectional causal relationship between obesity and osteoarthritis: Insights from a two-sample Mendelian randomization study
Source: Osteoarthr Cartil Open. 2025 May 27;7(3):100636. doi: 10.1016/j.ocarto.2025.100636 (PMC12226083; doi:10.1016/j.ocarto.2025.100636)

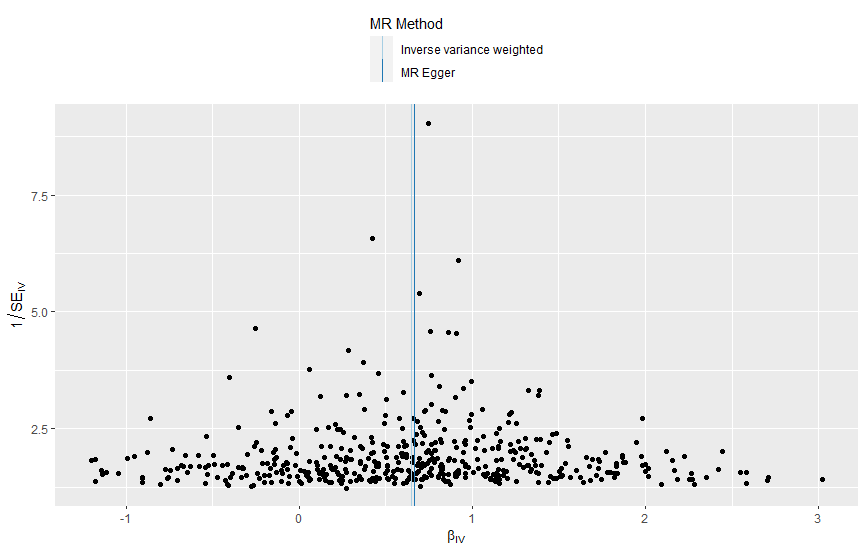
BMI to Knee OA


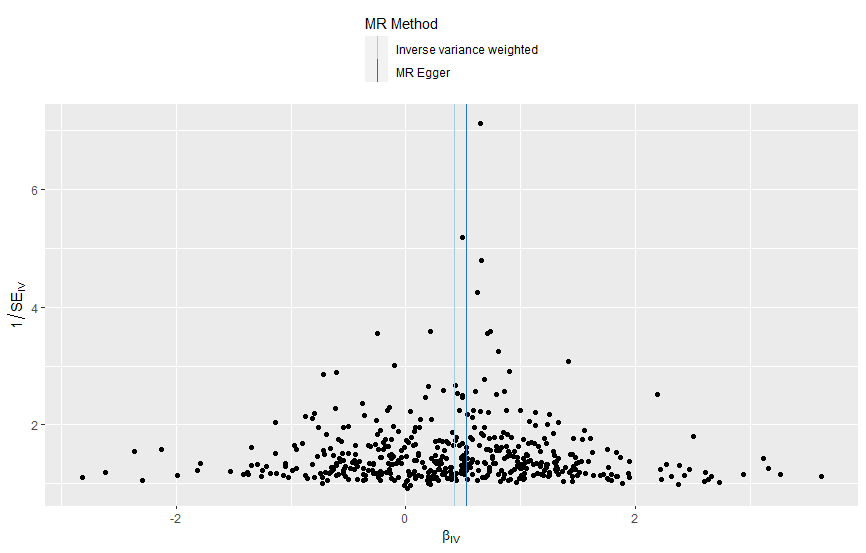
BMI to hip OA


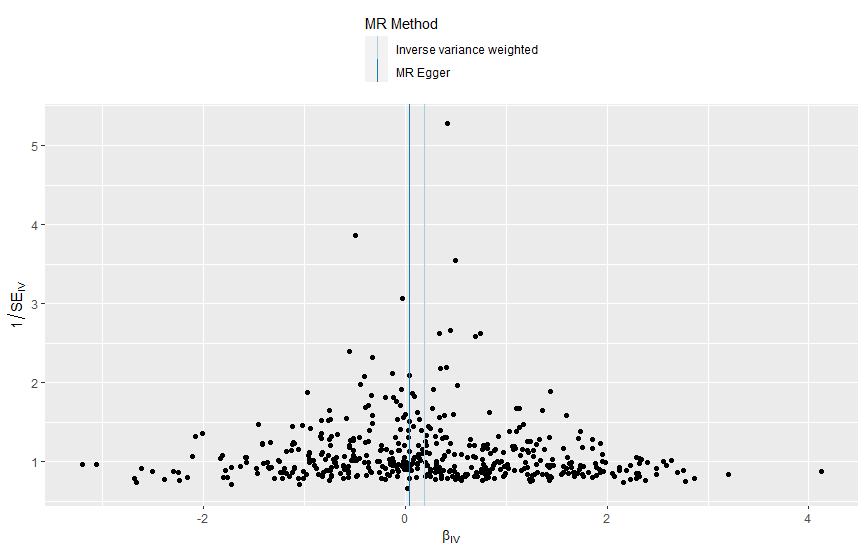
BMI to hand OA


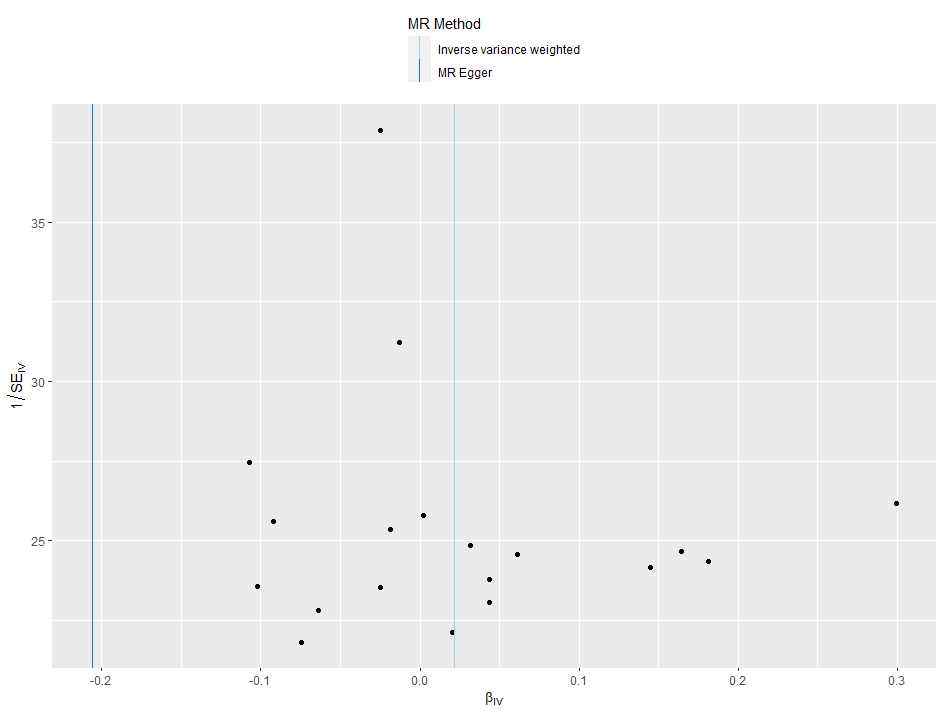
Knee OA to BMI

Hip OA to BMI


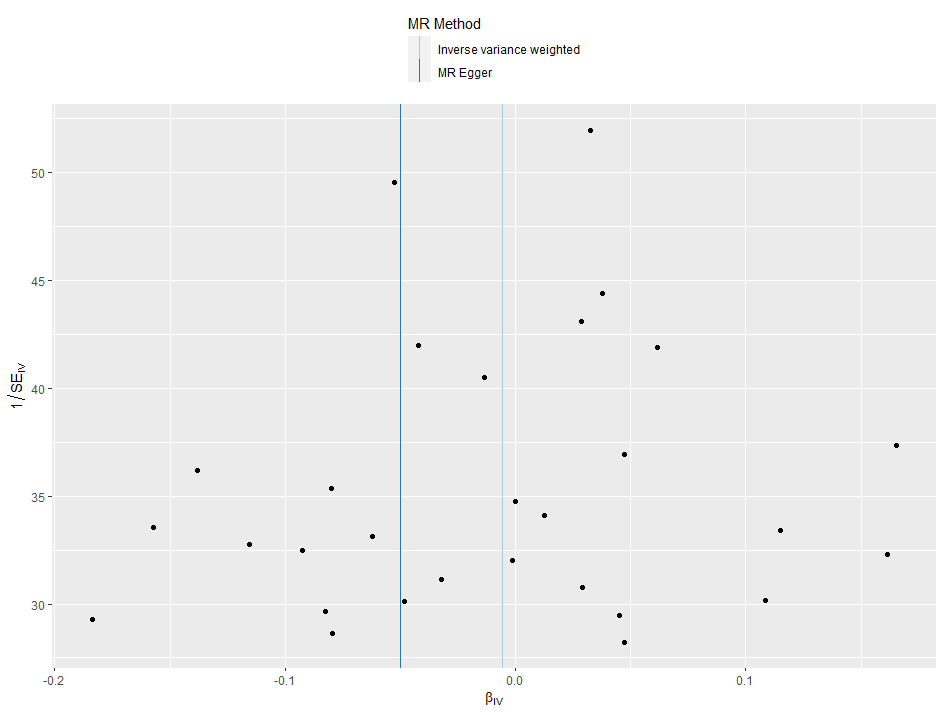


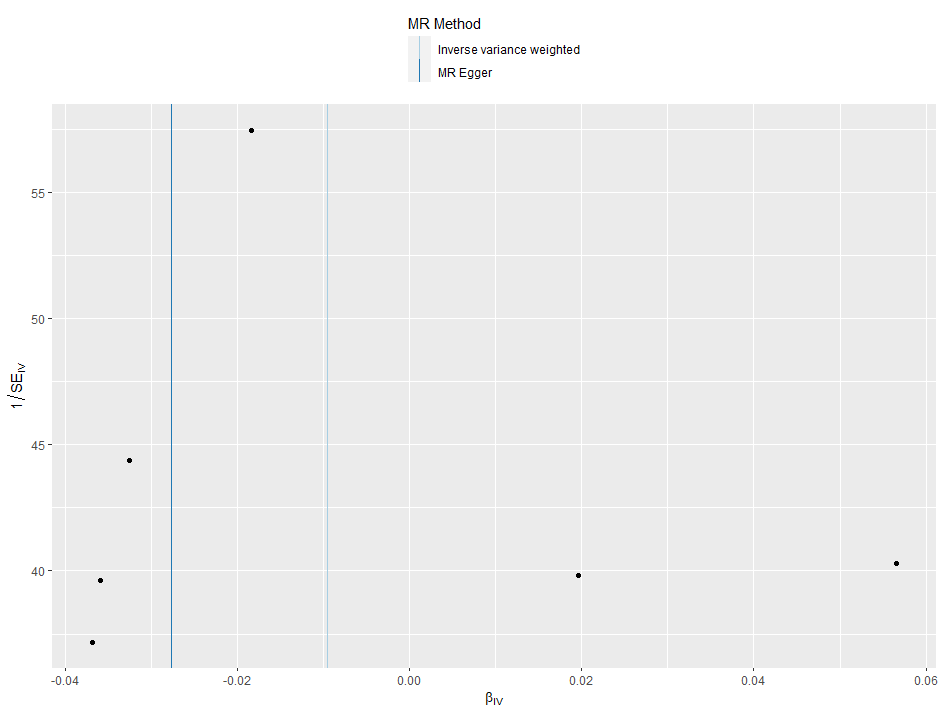
Hand OA to BMI

Supplement: Multimedia component 1 [file mmc1.docx]

BMI to Knee OA


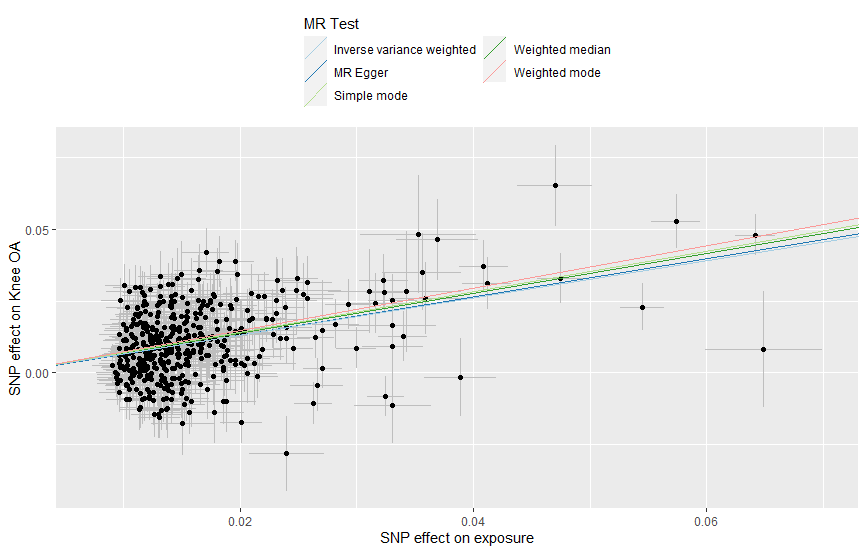


BMI to Hip OA


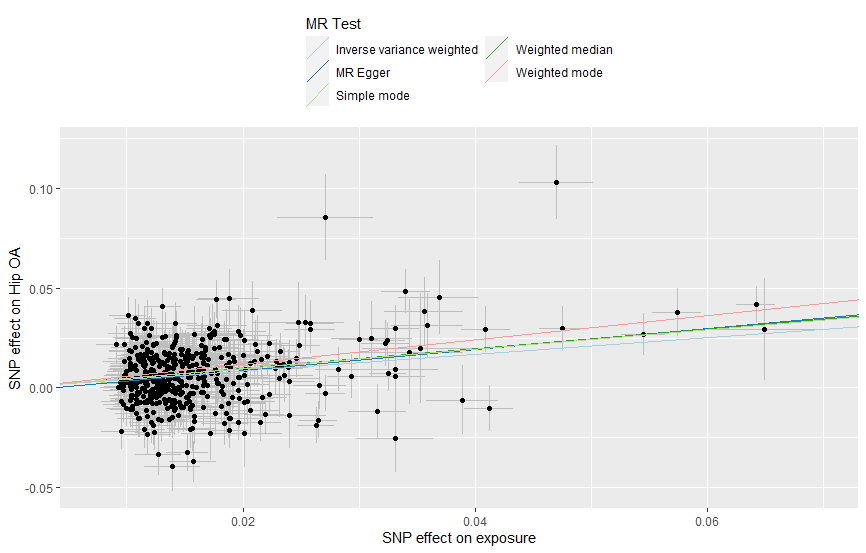


BMI to Hand OA


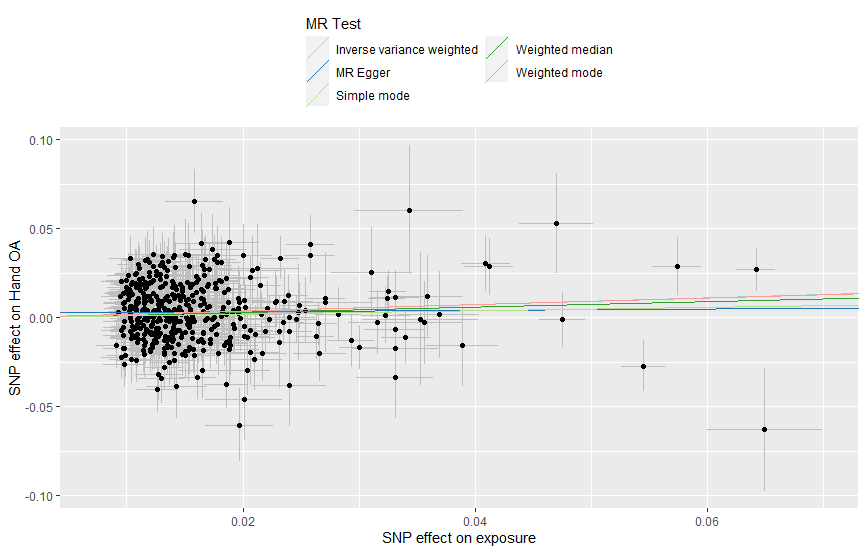


Knee OA to BMI


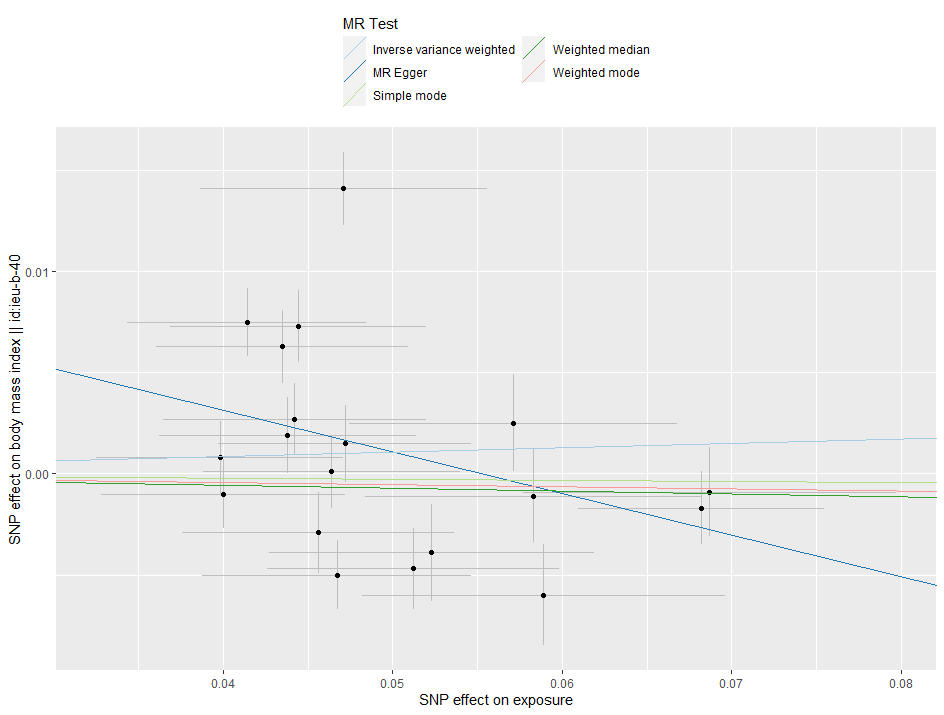


Hip OA to BMI


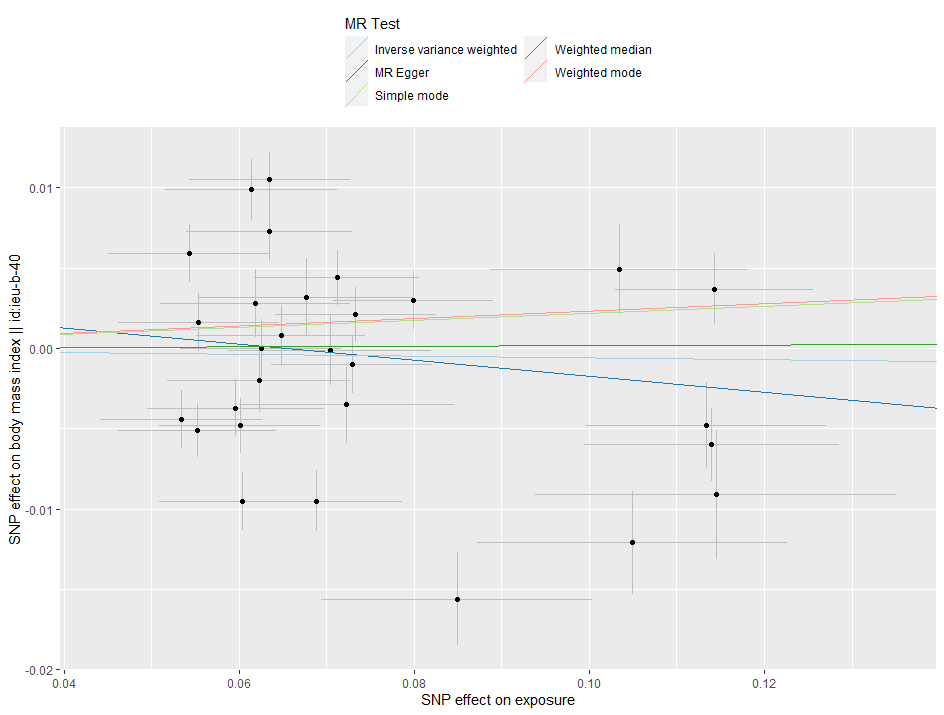


Hand OA to BMI


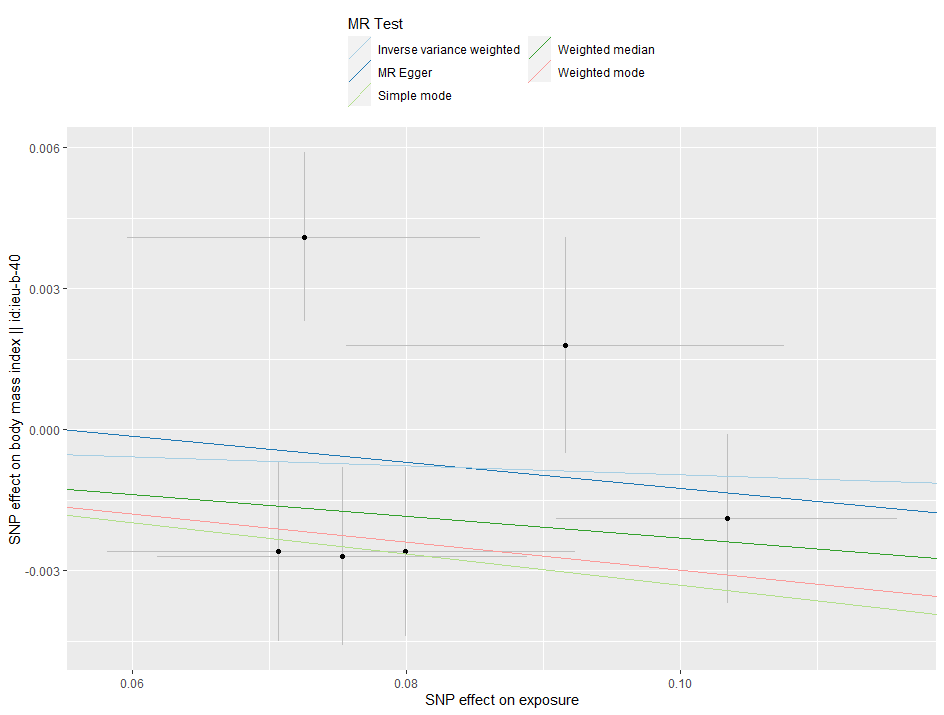

Supplement: Multimedia component 2 [file mmc2.docx]

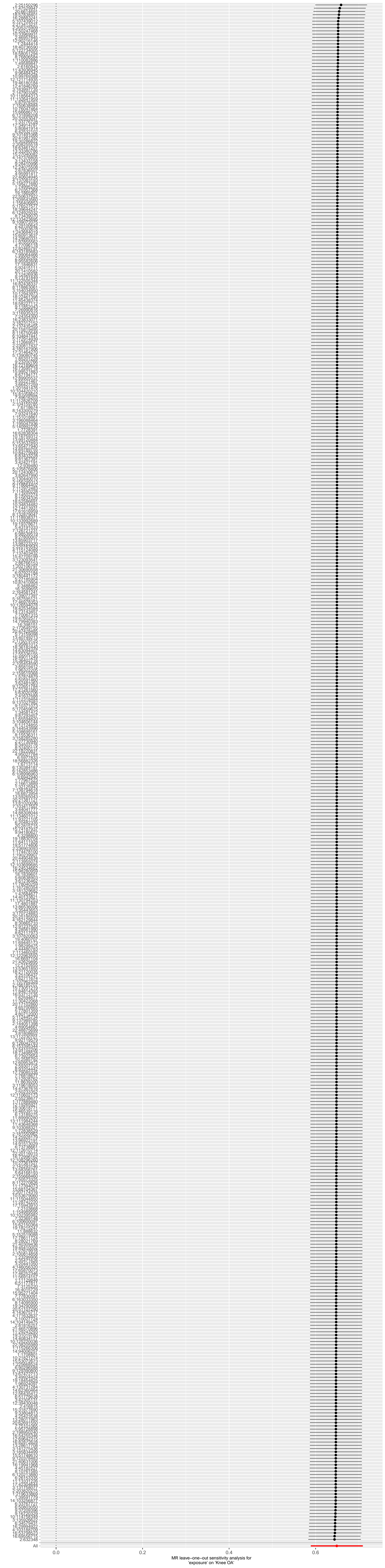

Supplement: Multimedia component 3 [file mmc3.pdf]

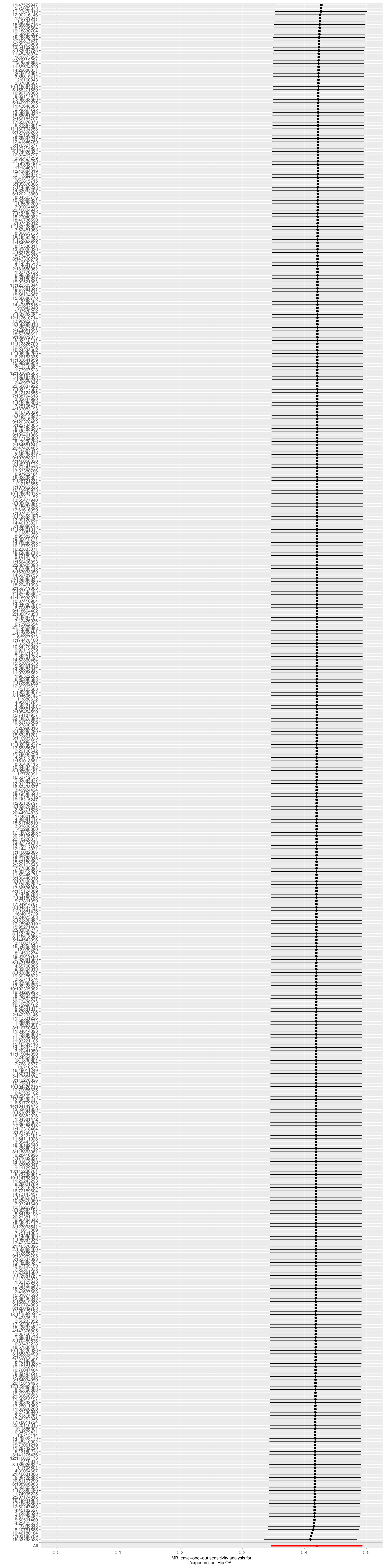

Supplement: Multimedia component 4 [file mmc4.pdf]

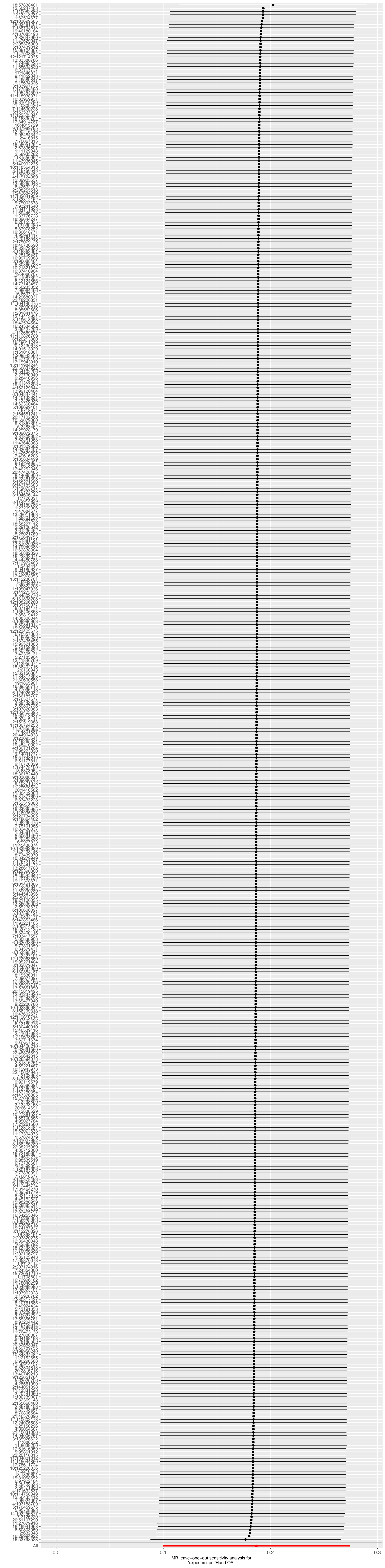

Supplement: Multimedia component 5 [file mmc5.pdf]

Knee OA to BMI


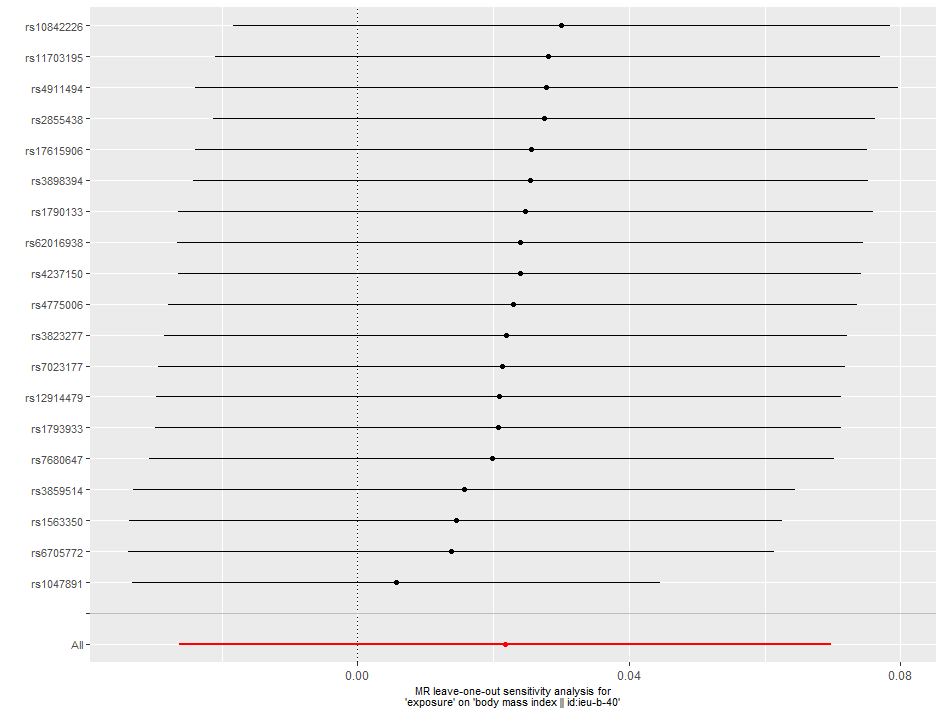


Hip OA to BMI


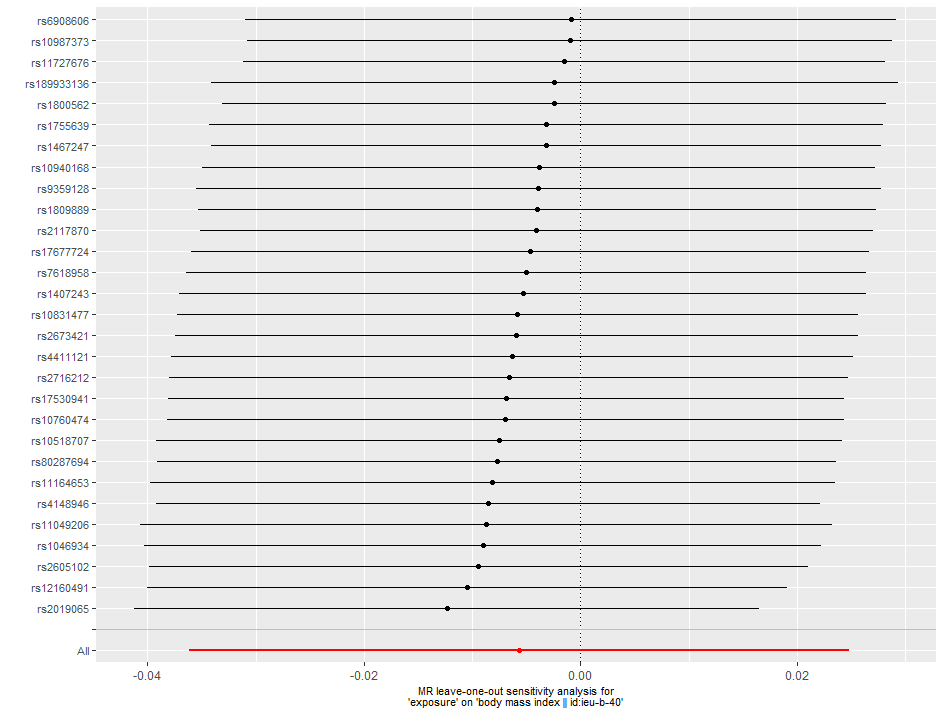


Hand OA to BMI


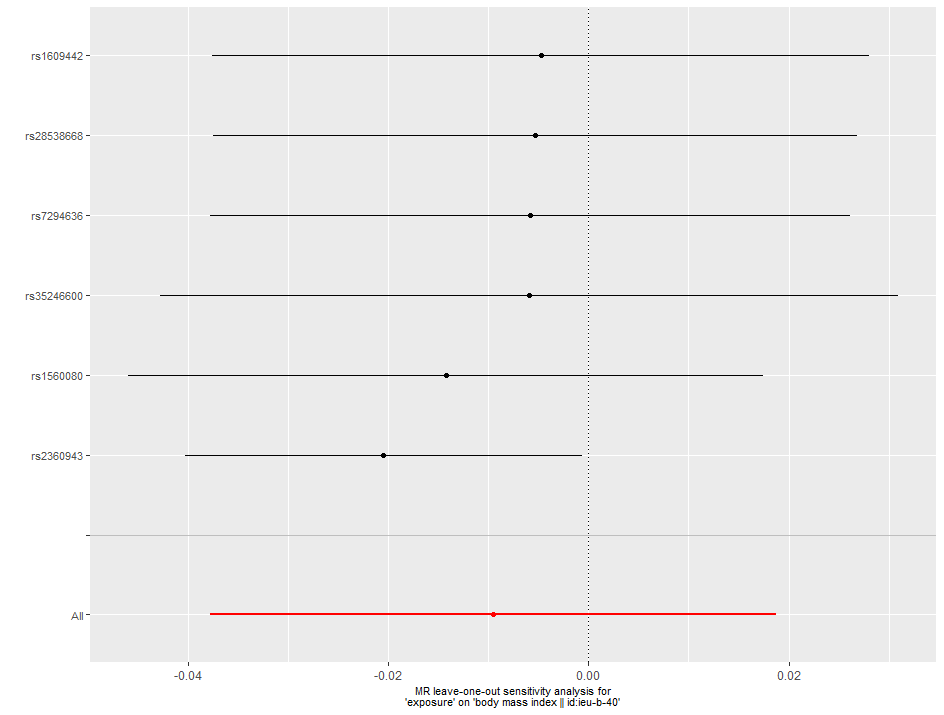

Supplement: Multimedia component 6 [file mmc6.docx]

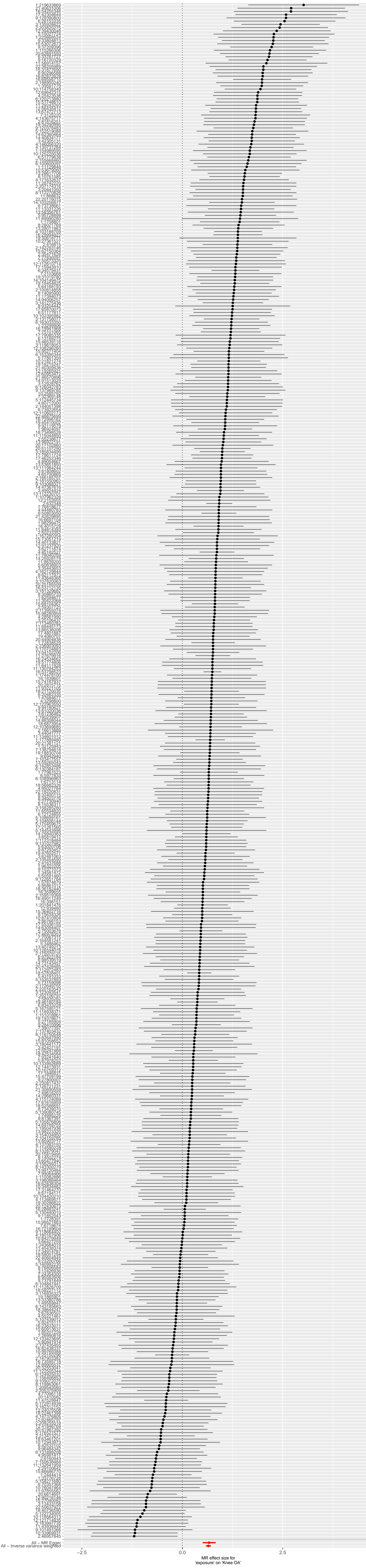

All - MR Egger  
All - Inverse variance weighted

MR effect size for  
'exposure' on 'Knee OA'

Supplement: Multimedia component 7 [file mmc7.pdf]

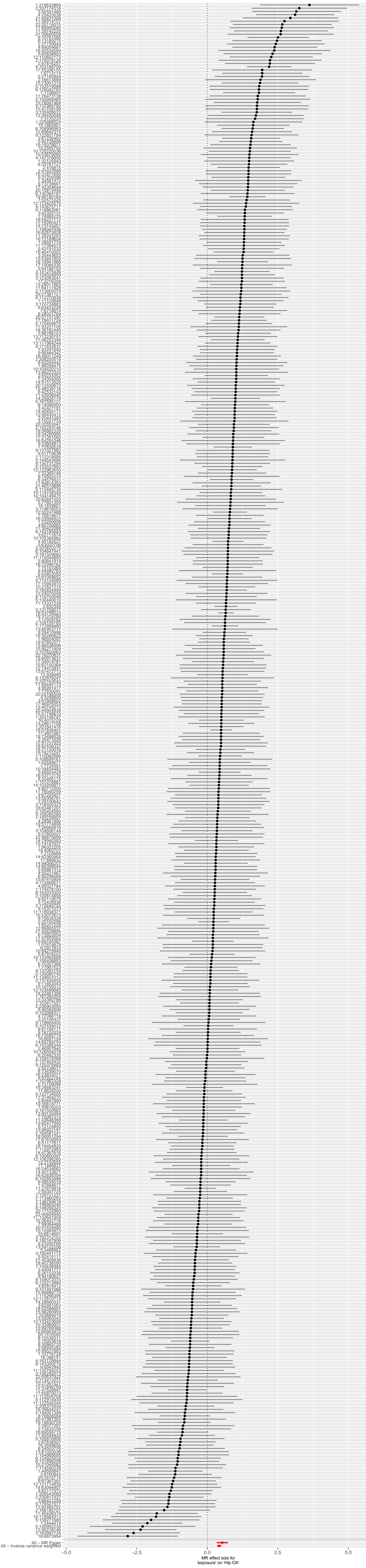

Supplement: Multimedia component 8 [file mmc8.pdf]

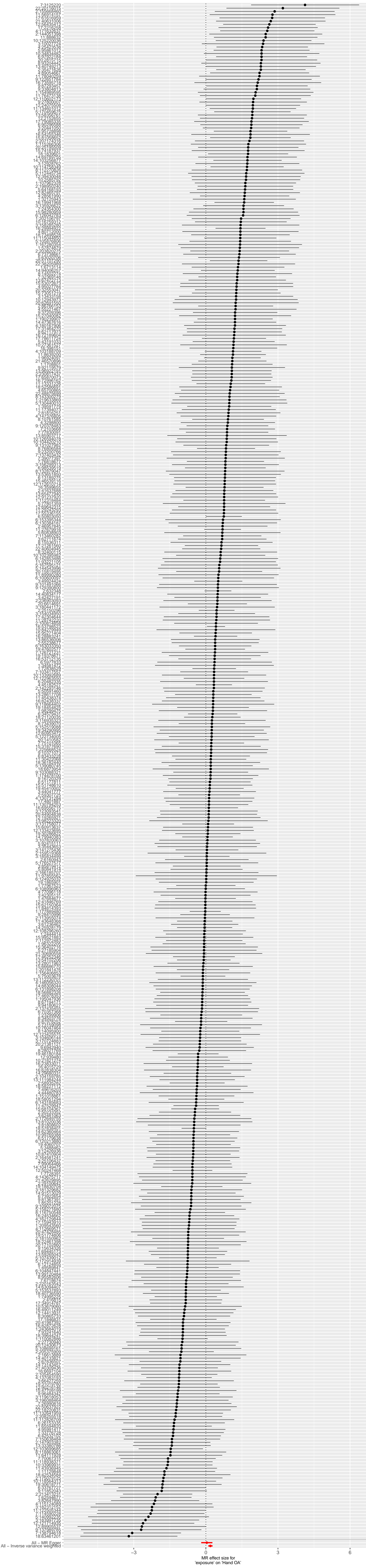

Supplement: Multimedia component 9 [file mmc9.pdf]

Knee OA to BMI


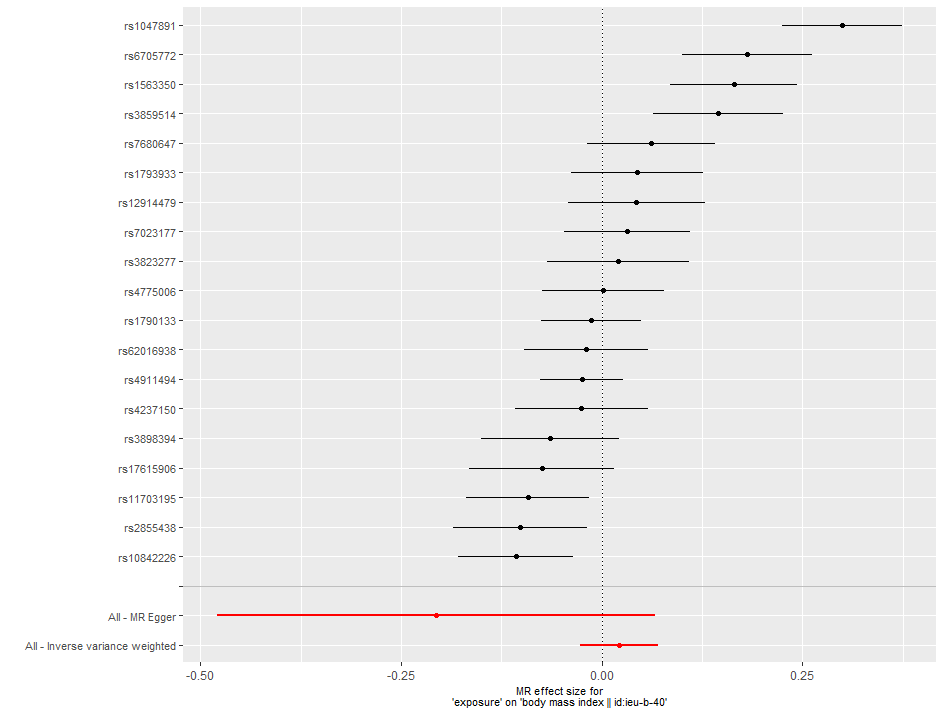


Hip OA to BMI


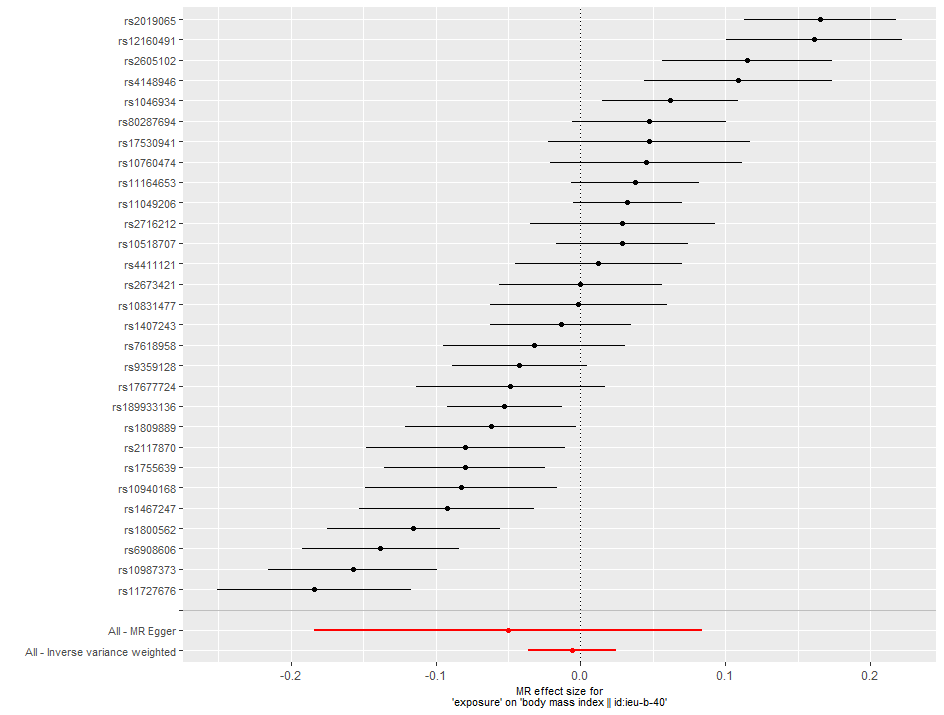


Hand OA to BMI


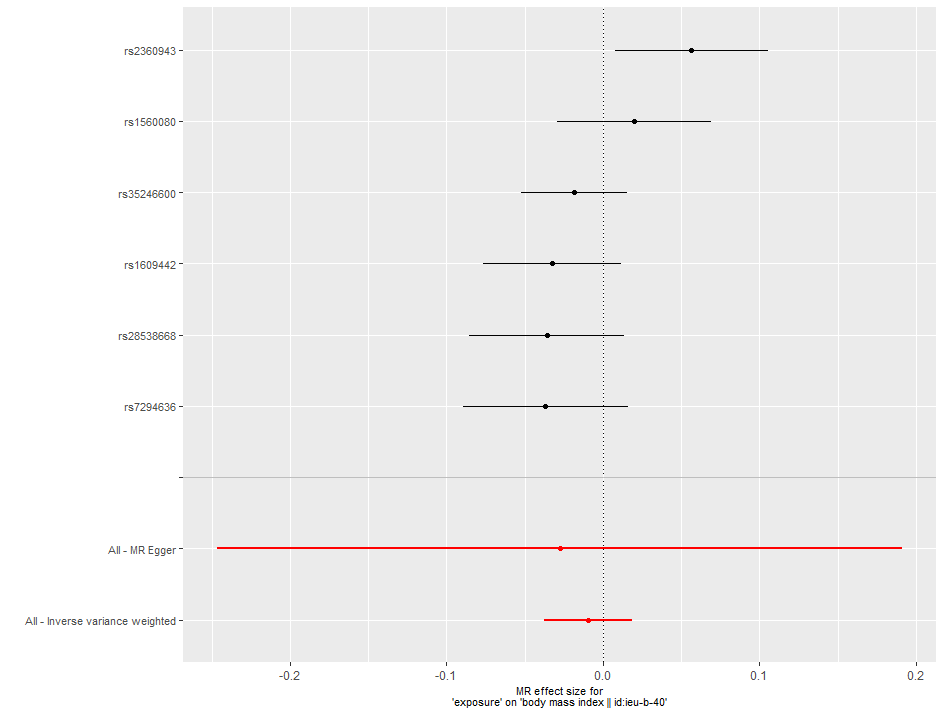

Supplement: Multimedia component 10 [file mmc10.docx]
